# Thermally Fully Developed Electroosmotic Flow of Power-Law Nanofluid in a Rectangular Microchannel

**DOI:** 10.3390/mi10060363

**Published:** 2019-05-30

**Authors:** Shuyan Deng

**Affiliations:** Institute of Architecture and Civil Engineering, Guangdong University of Petrochemical Technology, Maoming 525000, China; sydeng4-c@my.cityu.edu.hk; Tel.: +86-0668-273-7115

**Keywords:** power-law nanofluid, electroosmotic flow, heat transfer, compact difference scheme, nanoparticle volume friction, Joule heating parameter

## Abstract

The hydrodynamic and thermal behavior of the electroosmotic flow of power-law nanofluid is studied. A modified Cauchy momentum equation governing the hydrodynamic behavior of power-law nanofluid flow in a rectangular microchannel is firstly developed. To explore the thermal behavior of power-law nanofluid flow, the energy equation is developed, which is coupled to the velocity field. A numerical algorithm based on the Crank–Nicolson method and compact difference schemes is proposed, whereby the velocity, temperature, and Nusselt number are computed for different parameters. A larger nanoparticle volume fraction significantly reduces the velocity and enhances the temperature regardless of the base fluid rheology. The Nusselt number increases with the flow behavior index and with electrokinetic width when considering the surface heating effect, which decreases with the Joule heating parameter. The heat transfer rate of electroosmotic flow is enhanced for shear thickening nanofluids or at a greater nanoparticle volume fraction.

## 1. Introduction

The development of the micro-electric mechanical system (MEMS) has received great attention because of its innovative application in chemical, medical, and biological-related industries. For instance, Lab-on-a-chip, which conducts experiments by processing bioliquids or chemical solutions on a microchip, has become increasingly popular [1]. Electroosmotic flow (EOF) being a major electrokinetic effect refers to the motion of an ionized liquid inside microchannels with respect to the stationary charged channel walls under an external electric field applied tangentially along the microchannel [2]. An electroosmosis-based micropump has been successfully developed [3] and became one of the most important components in lab-on-a-chip since it has advantages, such as the production of pulse-free and plug-like flow, and the dependence on non-mechanical parts [4] over the conventional pressure-driven flow (PDF). Therefore, a fundamental understanding of the mechanism of EOF is vital for the precise control and optimal design of microfluidic devices.

The hydrodynamic behavior of EOF of different fluids is investigated in different microchannels, such as slit [5], rectangular [6,7], elliptic [8], and circular microchannels [9]. The core of the attention on EOF has shifted to the heat transfer characteristics of EOF [9,10,11], two-layer EOF [12], rotating EOF [13,14], and pressure effects on EOF [15]. In biological and chemical industries, biofluids, such as blood and DNA, solutions manipulated in microfluidic devices show nonlinear rheological behavior, such as the viscosity dependent on shear rate, which cannot be modeled by the linear constitutional relation. Therefore, the theory of non-Newtonian fluids offers researchers in the fields of mathematics and physics challenges in developing solutions to the nonlinear governing equations. The EOF of Jeffrey fluid [16] and Maxwell fluid [17] under an alternating current (AC) electric field are investigated in terms of the influence of nonlinear rheological behavior on flow performance. The corresponding analytical solution for the velocity field has been developed by adopting the method of variable separation. Das and Charkraborty [5] first proposed a non-Newtonian power-law model for the EOF of biofluids and acquired analytical solutions of velocity, temperature, and concentration distribution. The power-law model has received much more attention because of the concise expression and breadth coverage for working liquids in a MEMS. Zhao et al. highlighted the dynamic properties of EOF of power-law fluid under an AC electric field along a rectangular microchannel [18]. My previous works conducted parametric studies on the EOF of power-law fluids in terms of the flow behavior index and electrokinetic width in a cylindrical circular microcapillary [19] and in a rectangular microchannel [20]. It showed that the nonlinear rheological behavior of power-law fluid has a significant influence on the flow pattern and volumetric flow rate of EOF. Particularly, the shear thinning fluid needs more developed temporal length and is more sensitive to the change in parameters than that of shear thickening fluid.

The thermal characteristics have to be carefully considered due to the involvement with fluid flow and the applications in the cooling systems of a MEMS. The Joule heating effect that is an inherent phenomenon in EOF resulting from the ohmic resistance of the electrolyte dominates the heat generation of fluid flow. It results in the change of temperature-dependent electrical/transport properties, such as viscosity and electric conductivity of the working liquids, which, in turn, influences the hydrodynamic behavior of EOF. Concerning the Joule heating-induced heat transfer of EOF, the models in a microcapillary [9] and in a rectangular microchannel [21] without consideration of viscous dissipation have been developed. A model in a slit microchannel with consideration of viscous dissipation and thermal radiation is developed by Shit et al. [10], which has been extended to power-law fluid in a circular microchannel [22]. The entropy generation rate for the EOF of a power-law fluid in a microchannel has been obtained, and it turns out that entropy generation is dominated by the Joule heating effect [23]. In addition, the microchannel flow can be assisted by the mixed effects of electroosmosis and pressure gradient. The hydrodynamic and thermal behavior of combined EOF and PDF of Newtonian fluid in a microchannel was studied by solving the velocity distribution, temperature distribution, and Nusselt number [24]. The model above has been extended to the case of power-law fluid in a slit microchannel [25] and in a rectangular microchannel [26], where the temperature distribution and Nusselt number have been numerically solved by taking into account the Joule heating effect and viscous dissipation. Lately, the effects of magnetic field on the heat generation of EOF has been given importance [27].

Compared with the conventional fluid, the nanofluid containing nanoparticles with a diameter of 1 to 100 nm shows larger overall thermal conductivity. This is because the thermal conductivity of solid particles, such as Cu, is 700 times higher than the thermal conductivity of water, and the thermal conductivity of metal oxide, such as AI_2_O_3_, is much greater than that of a pure fluid, and hence, the thermal performance of nanofluid is dramatically enhanced in the presence of these nanoparticles. Furthermore, the nanofluid is stable, which has no additional problems like pressure drop, sedimentation, or blockage in microchannels. Choi firstly proposed the model of nanofluid [28]. In the fields of energy, power, electronics, etc., the trend for using suspensions of nanoparticles in a fluid medium is growing [29], which has led to several researches on the nanofluid flow [30,31]. The comparison among several existing models for thermal conductivity was carried out to assist in the accurate prediction of thermal behavior of nanofluid in [32]. A more comprehensive survey on the turbulent heat transfer of nanofluid flow was conducted [33]. Since the characteristics of nanofluid mentioned above meet the increasingly higher requirements in heat transfer of heat exchange equipment of MEMS, the nanofluid flow in microscale has been paid more and more attention recently. The electrokinetic effects of nanofluids induced by the streaming potential on PDF in microtubes were theoretically studied [34]. The transient thermal characteristics of the combined EOF and PDF of nanofluids in a microchannel under the effect of a magnetic field were investigated [35]. Furthermore, as heat transfer performance of fluid flow is increasingly encountered in micro electrics, the base fluid is not only limited to Newtonian fluid, but also the emphasis has been laid on the non-Newtonian fluid flow. Therefore, in microscale devices with characteristics of compact structure, to achieve the efficient thermal management of the non-Newtonian fluid, the power-law nanofluid was developed, where nanoparticles are added, and the base fluid rheology is described by the power-law model. The EOF of power-law nanofluid in a parallel plate microchannel was studied where one-dimensional momentum equation and energy equation were analytically solved [36]. A mixed convection flow and the corresponding heat transfer of shear thinning power-law nanofluid was respectively investigated by Ellahi et al. [37] and by Si et al. [38]. It turns out that the solid volume fractions exert special effects on both the velocity field and temperature field of fluid flow.

An up-to-date literature review indicates that for non-Newtonian fluids, the studies on the thermal behavior of EOF lack detailed insights into the substance of microstructure, namely the role that nanoparticles play, and the influence of channel geometry, owing to the complexity of governing equations for non-Newtonian nanofluid EOF in complex microchannels. Moreover, the thermal behavior of pure EOF of a power-law fluid in complex microchannels has not been studied yet. Due to the practical and fundamental significance of EOF of power-law nanofluid in a complex microchannel, such as a rectangular channel, and motivated by the works reviewed above, it is necessary to provide a fundamental understanding of the Joule heating induced heat transfer characteristics. This paper develops a model for thermally fully developed EOF of power-law nanofluid in a rectangular microchannel. The viscosity of power-law nanofluid depends both on the flow behavior index and the volume fraction of nanoparticles. By numerically solving the modified momentum equation and the energy equation, the velocity distribution, temperature distribution, and Nusselt number under the influence of the Joule heating effect are evaluated for different nanoparticle volume fraction, electrokinetic width, flow behavior index. The results are useful for the optimal design and active control of microfluidic systems.

## 2. Mathematical Modeling

**Figure 1:**
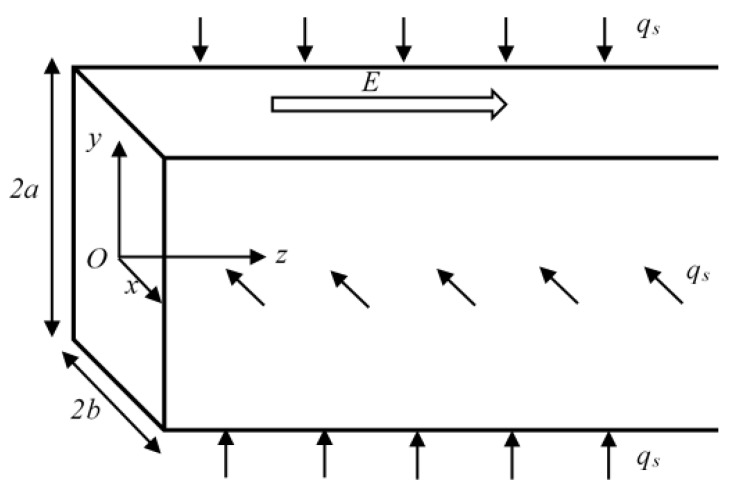
Sketch of the rectangular microchannel with height 2*a*, width 2*b*, and constant heat flux *q_s_* at the walls.

As sketched in Figure 1, a rectangular microchannel of width 2*b* and height 2*a* is considered. The channel is filled with power-law nanofluid with a dielectric constant *ε* and is subject to constant heat flux *q_s_*. The channel wall is uniformly charged with zeta potential *ξ,* and the electric double layers (EDLs) will not overlap. As an electric field is applied tangentially along the channel (*z*-direction), the liquid inside is set in motion due to the existence of free ions in EDLs. Owing to the symmetry, the following analysis is limited to a quarter of the channel, Ω, and the coordinate system used is shown in Figure 1.

### 2.1. Electric Potential Distribution

Based on the electrostatics theory, the relationship between the electric potential distribution *ψ*(*x*, *y*) and the local net charge density *ρ_e_* is expressed by the Poisson equation.

(1) $$\frac{\partial^{2}\psi }{\partial x^{2}}+\frac{\partial^{2}\psi }{\partial y^{2}}=-\frac{\rho_{e}}{\varepsilon \varepsilon_{0}}.$$

The local net charge density *ρ_e_* is subject to Boltzmann distribution.

(2) $$\rho_{e}=-2\chi en_{0}\sinh\left(\frac{\chi e\psi }{k_{b}T_{0}}\right),$$

where *χ* denotes the valence of ions, *e* denotes the elementary charge, *n*_0_ is the ion density of the bulk liquid, *k_b_* is the Boltzmann constant, and *T*_0_ implies the absolute temperature. Substituting Equation (2) to Equation (1) yields the two-dimensional Poisson–Boltzmann (P-B) equation for the electric potential distribution of EDL in the rectangular microchannel,

(3) $$\frac{\partial^{2}\psi }{\partial x^{2}}+\frac{\partial^{2}\psi }{\partial y^{2}}=\frac{2\chi en_{0}}{\varepsilon }\sinh\left(\frac{\chi e\psi }{k_{b}T_{0}}\right).$$

The corresponding boundary conditions are given as

*∂ψ/∂x*| _*x* = 0_ = 0*, ∂ψ/∂y*|_*y* = 0_ = 0, *ψ*| _*x = b*_ = *ξ, ψ*|_*y = a*_ = *ξ*. (4)

To facilitate the discussion, the following scales are introduced $\bar{x}=x/a$, $\bar{y}=y/a$, $\bar{\xi }=\chi e\xi /(k_{b}T_{0})$, *α = b/a*, K=κa, with *K* defined as the dimensionless electrokinetic width and 1/κ as the thickness of EDL, where κ = [2*χe*^2^*n*_0_/(*εk_b_T*_0_)]^1/2^. Accordingly, the dimensionless P-B equation and the boundary conditions are obtained

(5) $$\frac{\partial^{2}\bar{\psi }}{\partial {\bar{x}}^{2}}+\frac{\partial^{2}\bar{\psi }}{\partial {\bar{y}}^{2}}=K^{2}\sinh(\bar{\psi })$$

(6) $$\partial \bar{\psi }/\partial \bar{x}{|}_{\bar{x}=0}=0,\partial \bar{\psi }/\partial \bar{y}{|}_{\bar{y}=0}=0,\;\bar{\psi }{|}_{\bar{x}=\alpha }=\bar{\xi },\;\bar{\psi }{|}_{\bar{y}=1}=\bar{\xi }.$$

For small zeta potential (*ξ* ≤ 0.025 V), the known Debye–Hückel linearization principle [5] $\sinh(\bar{\psi })\approx \bar{\psi }$ is used to linearize the P-B equation. In this case, an analytical solution to Equations (5) and (6) can be readily solved by using variable separation method as

$$\begin{array}{l} \bar{\psi }(\bar{x},\bar{y})=2\bar{\xi }\sum_{M=1}^{\infty }\frac{{\left(-1\right)}^{M+1}\cos(\mu_{M}\bar{y})\cosh(\sqrt{\mu_{M}^{2}+K^{2}}\bar{x})}{\mu_{M}\cosh(\sqrt{\mu_{M}^{2}+K^{2}}\alpha )}\\ +2\bar{\xi }\sum_{N=1}^{\infty }\frac{{\left(-1\right)}^{N+1}\cos(\sigma_{N}\bar{x})\cosh(\sqrt{\sigma_{N}^{2}+K^{2}}\bar{y})}{\alpha \sigma_{N}\cosh(\sqrt{\sigma_{N}^{2}+K^{2}})} \end{array}$$

where $\mu_{M}=(2M-1)\pi /2$, $\sigma_{N}=(2N-1)\pi /(2\alpha )$, *M*,*N* = 1,2,3,…

### 2.2. Velocity Distribution

The vector formation of Cauchy momentum equation reads

(7) $$\rho \left(\frac{\partial \vec{V}}{\partial t}+\vec{V}\cdot \nabla \vec{V}\right)=-\nabla p+\nabla \cdot \vec{\tau }+\vec{E}\rho_{e}$$

where $\vec{V}$ is the velocity field of EOF, *ρ* represents the density of mass, *t* denotes the variable of time, ∇p is the pressure gradient, $\vec{E}$ is the electric field strength, $\dot{\tau }=2\mu_{eff}\dot{\gamma }\vec{e}$ denotes the stress tensor which can be expressed by a second invariant of the strain rate tensor *e_ij_* = [(*∂u_i_/∂x_j_*) + (*∂u_j_/∂x_i_*)]/2 as $\dot{\gamma }={\left(2e_{kl}e_{kl}\right)}^{1/2}$. *μ_eff_* denotes the effective viscosity coefficient. For a fully developed, incompressible and laminar EOF of power-law nanofluid, it is assumed that there is only an axial velocity *w* = *w*(*x, y)* [7,20], accordingly, the effective viscosity of the power-law nanofluid is *μ_eff_ = μ_f_/*(*1 − ϕ*)^5/2^. The viscosity of the base fluid is *μ_f_ = η*(*|∂w/∂x|^n − 1^, |∂w/∂y|^n − 1^*) which is a function of strain rate, flow consistency index *η* of dimension (Nm^−2^s^n^) [7,20], and volume fraction of nanoparticle *ϕ* [39]. Finally, one can express the constitutive equation of power-law nanofluid as

(8) $$\tau =\mu_{eff}\cdot \dot{\gamma }=\frac{\eta }{{\left(1-\phi \right)}^{5/2}}\left({\left|\frac{\partial w}{\partial x}\right|}^{n-1}\frac{\partial w}{\partial x}+{\left|\frac{\partial w}{\partial y}\right|}^{n-1}\frac{\partial w}{\partial y}\right),$$

where τ is the shear stress, and *n* is the flow behavior index. *n* = 1 represents a Newtonian nanofluid, *n* < 1 and *n* > 1 represents the shear thinning nanofluids (pseudoplastic nanofluids) and the shear thickening nanofluids (dilatant nanofluids), respectively. Consequently, the modified Cauchy momentum equation for power-law nanofluid EOF is

(9) $$\frac{n\eta }{{\left(1-\phi \right)}^{5/2}}\left({\left|\frac{\partial w}{\partial x}\right|}^{n-1}\frac{\partial^{2}w}{\partial x^{2}}+{\left|\frac{\partial w}{\partial y}\right|}^{n-1}\frac{\partial^{2}w}{\partial y^{2}}\right)+E\rho_{e}=0.$$

The corresponding boundary conditions are

(10) $$\partial w/\partial x{|}_{x=0}=0,\partial w/\partial y{|}_{y=0}=0,w{|}_{x=b}=0,w{|}_{y=a}=0.$$

Introducing the following scales $\bar{w}=w/W$ with $W=-\varepsilon k_{b}ET_{0}/(\mu_{0}\chi e)$ where *μ*_0_ is the viscosity coefficient of Newtonian fluid of dimension (Nm^−2^s), one can obtain the dimensionless modified Cauchy momentum equation and the boundary conditions:

(11) $$\frac{n\eta }{\mu_{0}{\left(1-\phi \right)}^{5/2}}{\left(\frac{W}{a}\right)}^{n-1}\left({\left|\frac{\partial \bar{w}}{\partial \bar{x}}\right|}^{n-1}\frac{\partial^{2}\bar{w}}{\partial {\bar{x}}^{2}}+{\left|\frac{\partial \bar{w}}{\partial \bar{y}}\right|}^{n-1}\frac{\partial^{2}\bar{w}}{\partial {\bar{y}}^{2}}\right)+K^{2}\sinh(\bar{\psi })=0,$$

(12) $$\partial \bar{w}/\partial \bar{x}{|}_{\bar{x}=0}=0,\partial \bar{w}/\partial \bar{y}{|}_{\bar{y}=0}=0,\bar{w}{|}_{\bar{x}=\alpha }=0,\bar{w}{|}_{\bar{y}=1}=0.$$

When considering Newtonian nanofluid, i.e., *n* = 1, and using the known Debye–Hückel principle, an analytical solution can be obtained by the method of variable separation and the method of constant variation [40], which is expressed as

(13) $$\bar{w}(\bar{x},\bar{y})=\sum_{I=1}^{\infty }\cos(\sqrt{\beta_{I}}\bar{x})U_{I}(\bar{y}),$$

where $\beta_{I}=(2I-1)\pi /(2\alpha )$ with I = 1,2,3,…, $A=-\frac{8}{\alpha }K^{2}{\left(1-\phi \right)}^{5/2}\bar{\xi }$,

$U_{I}(\bar{y})=-\frac{A}{C_{I}\cosh(C_{I})}\left(\sum_{M=1}^{\infty }\frac{A_{MI}G_{MI}}{D_{M}}+\sum_{N=1}^{\infty }\frac{B_{NI}Q_{NI}}{E_{N}}\right)\cosh(C_{I}\bar{y})$,

$+A\left\{\sum_{M=1}^{\infty }\frac{A_{MI}[\cosh(C_{I}\bar{y})-\cos(\mu_{M}y)]}{D_{M}(\mu_{M}{}^{2}+C_{I}{}^{2})}+\sum_{N=1}^{\infty }\frac{B_{NI}[\cosh(\sqrt{\sigma_{N}{}^{2}+K^{2}}y)-\cosh(C_{I}\bar{y})]}{E_{N}(\sigma_{N}{}^{2}+K^{2}-C_{I}{}^{2})}\right\}$,

$A_{MI}=\frac{\left[\sqrt{\mu_{M}^{2}+K^{2}}\sinh(\sqrt{\mu_{M}^{2}+K^{2}}\alpha )\cos(\sqrt{\beta_{I}}\alpha )+\sqrt{\beta_{I}}\cosh(\sqrt{\mu_{M}^{2}+K^{2}}\alpha )\sin(\sqrt{\beta_{I}}\alpha )\right]}{\mu_{M}^{2}+K^{2}+\beta_{I}}$,

$B_{NI}=\frac{1}{2}\left\{\frac{\sin[(\sigma_{N}^{}+\sqrt{\beta_{I}})\alpha ]}{\sigma_{N}^{}+\sqrt{\beta_{I}}}+\frac{\sin[(\sigma_{N}^{}-\sqrt{\beta_{I}})\alpha ]}{\sigma_{N}^{}-\sqrt{\beta_{I}}}\right\}$, $C_{I}{}^{2}=\beta_{I}$$D_{M}={\left(-1\right)}^{M+1}(2M-1)\pi \cosh(\sqrt{\mu_{M}^{2}+K^{2}}\alpha )$, $E_{N}={\left(-1\right)}^{N+1}(2N-1)\pi \cosh(\sqrt{\sigma_{N}^{2}+K^{2}})$, $G_{MI}=\frac{C_{I}[\cosh(C_{I})-\cos(\mu_{M})]}{\mu_{M}{}^{2}+C_{I}{}^{2}}$,

$Q_{NI}=\frac{C_{I}[\cosh(\sqrt{\sigma_{N}{}^{2}+K^{2}})-\cosh(C_{I})]}{\sigma_{N}{}^{2}+K^{2}-C_{I}{}^{2}}$.

In the case of non-Newtonian power-law nanofluid flow (*n* ≠ 1), a numerical algorithm is proposed to obtain the velocity field, which is presented in the next section.

### 2.3. Temperature Distribution

Assuming the thermophysical properties are constant, the heat generation induced by the viscous dissipation is much smaller than that induced by Joule heating [23,41] and thus, is neglected in the present analysis. The energy equation for the flow field in a rectangular microchannel is

(14) $${\left(\rho c_{p}\right)}_{eff}w\frac{\partial T}{\partial z}=k_{eff}\nabla^{2}T+\sigma E^{2}$$

with ${\left(\rho c_{p}\right)}_{eff}=\phi {\left(\rho c_{p}\right)}_{s}+(1-\phi ){\left(\rho c_{p}\right)}_{f}$ and $k_{eff}=\frac{k_{s}+2k_{f}+2(k_{s}-k_{f}){\left(1+\omega \right)}^{3}\phi }{k_{s}+2k_{f}-2(k_{s}-k_{f}){\left(1+\omega \right)}^{3}\phi }k_{f}$ [32,42]. *T* denotes temperature field, *ω* implies the ratio of the nanolayer thickness to the original particle radius. *σ, k*, and (*ρc_p_*) denote electrical conductivity, thermal conductivity, and heat capacity of power-law nanofluid at the reference pressure, respectively. The subscripts *s*, *f*, and *eff* denote the solid nanoparticles, base fluid, and nanofluid, respectively. The second term at the right-hand side of Equation (13) denotes the volumetric heat generation owing to the Joule heating effect. The corresponding boundary conditions are

(15) $$\partial T/\partial x{|}_{x=0}=0,\partial T/\partial y{|}_{y=0}=0,\partial T/\partial x{|}_{x=b}=q_{s}/k_{eff},\partial T/\partial y{|}_{y=a}=q_{s}/k_{eff}\;(\mathrm{or}\;T{|}_{y=a}=T_{w}(z)),$$

where *q_s_ = h*(*T_w_ − T_m_*) implies the constant wall heat flux and *h* is the convective heat transfer coefficient. Subscripts *w* and *m* denote the local wall value and mean value, respectively. As assumed in the preceding section, for thermally fully developed EOF, one has $\partial T/\partial z=dT_{m}/dz=dT_{w}/dz=0$ and thus $\partial^{2}T/\partial z^{2}=0$. It is noted that the temperature variation with respect to *z*-direction cannot be neglected and thus, the temperature on the wall is a function of *z*. Plus, an overall energy balance at an elemental control volume on a length of duct *dz* gives:

(16) $$\frac{dT_{m}}{dz}=\frac{q_{s}(a+b)+\sigma E^{2}ab}{{\left(\rho c_{p}\right)}_{eff}ab{\bar{w}}_{m}}$$

with the mean velocity ${\bar{w}}_{m}=w_{m}/W=\frac{1}{\alpha }\int_{0}^{\alpha }\int_{0}^{1}\bar{w}d\bar{x}d\bar{y}$.

Introducing $\bar{T}=k_{f}(T-T_{w})/(q_{s}a)$ and the Joule heating parameter $S=\sigma E^{2}a/q_{s}$, namely, the ratio of Joule heating to the heat flux from the channel wall, the dimensionless energy equation and the corresponding boundary conditions are obtained as

(17) $$\frac{\partial^{2}\bar{T}}{\partial {\bar{x}}^{2}}+\frac{\partial^{2}\bar{T}}{\partial {\bar{y}}^{2}}=\frac{k_{f}}{k_{eff}}\left[\left(1+\frac{1}{\alpha }+S\right)\frac{\bar{w}}{{\bar{w}}_{m}}-S\right],$$

(18) $$\partial \bar{T}/\partial \bar{x}{|}_{\bar{x}=0}=0,\;\partial \bar{T}/\partial \bar{y}{|}_{\bar{y}=0}=0,\;\partial \bar{T}/\partial \bar{x}{|}_{\bar{x}=\alpha }=k_{f}/k_{eff},\partial \bar{T}/\partial \bar{y}{|}_{\bar{y}=1}=k_{f}/k_{eff},(\mathrm{or}\;\bar{T}{|}_{\bar{y}=1}=0).$$

In the case of Newtonian fluid and using the known Debye–Hückel linearization principle, an analytical solution of temperature distribution is obtained by the method of variable separation and the method of constant variation,

(19) $$\bar{T}(\bar{x},\bar{y})=\sum_{I=1}^{\infty }\cos(\sqrt{\beta_{I}}\bar{x})Y_{I}(\bar{y}),$$

where $Y_{I}(\bar{y})=-L_{I}^{\left(0\right)}\{\frac{-k_{1}AL_{I}{}^{\left(1\right)}}{L_{I}^{\left(0\right)}}\left[\sum_{M=1}^{\infty }\frac{A_{MI}G_{MI}}{D_{M}}+\sum_{N=1}^{\infty }\frac{B_{NI}Q_{NI}}{E_{N}}\right]$

$+k_{1}A\sum_{M=1}^{\infty }\frac{A_{MI}(L_{I}{}^{\left(1\right)}-L_{MI}{}^{\left(2\right)})}{D_{M}(\mu_{M}{}^{2}+C_{I}{}^{2})}$ $+k_{1}A\sum_{N=1}^{\infty }\frac{B_{NI}(L_{NI}{}^{\left(3\right)}-L_{I}{}^{\left(1\right)})}{E_{N}(\sigma_{N}{}^{2}+K^{2}-C_{I}{}^{2})}$ $+\frac{B_{I}}{C_{I}}\left(1-\cosh C_{I}\right)\}\cosh(C_{I}\bar{y})$

$+\frac{B_{I}}{C_{I}{}^{2}}[1-\cosh(C_{I}\bar{y})]$ $-\frac{k_{1}A}{C_{I}L_{I}^{\left(0\right)}}\left(\sum_{M=1}^{\infty }\frac{A_{MI}G_{MI}}{D_{M}}+\sum_{N=1}^{\infty }\frac{B_{NI}Q_{NI}}{E_{N}}\right)\frac{\bar{y}\sinh(C_{I}\bar{y})}{2}$

$+\frac{k_{1}A}{C_{I}}\sum_{M=1}^{\infty }\frac{A_{MI}}{D_{M}(\mu_{M}{}^{2}+C_{I}{}^{2})}\left\{\frac{\bar{y}\sinh(C_{I}\bar{y})}{2}+\frac{C_{I}[\cos(\mu_{M}\bar{y})-\cosh(C_{I}\bar{y})]}{C_{I}{}^{2}+\mu_{M}{}^{2}}\right\}$

$+\frac{k_{1}A}{C_{I}}\sum_{N=1}^{\infty }\frac{B_{NI}}{E_{N}(\sigma_{N}{}^{2}+K^{2}-C_{I}{}^{2})}\left\{\frac{C_{I}[\cosh(\sqrt{\sigma_{N}{}^{2}+K^{2}}\bar{y})-\cosh(C_{I}\bar{y})]}{\sigma_{N}{}^{2}+K^{2}-C_{I}{}^{2}}-\frac{\bar{y}\sinh(C_{I}\bar{y})}{2}\right\}$,

$L_{I}{}^{\left(0\right)}=\frac{1}{C_{I}\cosh(C_{I})}$, $L_{I}{}^{\left(1\right)}=\frac{1}{2}\sinh(C_{I})$, $L_{MI}{}^{\left(2\right)}=-\frac{C_{I}[\cos(\mu_{M})-\cosh(C_{I})]}{C_{I}{}^{2}+\mu_{M}{}^{2}}$, and

$L_{NI}{}^{\left(3\right)}=\frac{C_{I}[\cosh(\sqrt{\sigma_{N}{}^{2}+K^{2}})-\cosh(C_{I})]}{\sigma_{N}{}^{2}+K^{2}-C_{I}{}^{2}}$.

The detailed procedure is presented in Appendix A.

For non-Newtonian power-law nanofluid, the energy equation can be solved using the numerical algorithm developed later.

### 2.4. Entropy Generation Analysis

With the temperature distribution obtained, Nusselt number indicating heat transfer rate from the channel wall to the liquid inside the channel is deduced as:

(20) $$Nu=\frac{ha}{k_{eff}}=-\frac{k_{f}}{k_{eff}{\bar{T}}_{m}},$$

where the mean temperature is

(21) $${\bar{T}}_{m}=\frac{\int_{0}^{1}\int_{0}^{\alpha }\bar{T}\bar{w}d\bar{x}d\bar{y}}{\int_{0}^{1}\int_{0}^{\alpha }\bar{w}d\bar{x}d\bar{y}}=\frac{k_{f}(T_{m}-T_{w})}{q_{s}a}$$

and the convective heat transfer coefficient is *h = q_s_*/(*T_w_ − T_m_*).

The entropy generation rate per unit volume is presented, which evaluates the amount of useful energy destroyed during the process, namely, the thermodynamic irreversibility of EOF [35,43,44].

(22) $$S_{L}=S_{H}+S_{J}=\frac{k_{eff}}{T^{2}}\left(\frac{\partial^{2}T}{\partial x^{2}}+\frac{\partial^{2}T}{\partial y^{2}}\right)+\frac{\sigma E^{2}}{T}.$$

Since the heat generated by viscous dissipation is negligible [23,41], the irreversibility of the system equals the summation of local volumetric entropy generation rate due to heat transfer and Joule heating. The corresponding nondimensional form is

(23) $${\bar{S}}_{L}={\bar{S}}_{H}+{\bar{S}}_{J}=\frac{k_{eff}}{k_{f}{\left(\bar{T}+\Theta \right)}^{2}}\left[{\left(\frac{\partial \bar{T}}{\partial \bar{x}}\right)}^{2}+{\left(\frac{\partial \bar{T}}{\partial \bar{y}}\right)}^{2}\right]+\frac{SE^{2}}{\bar{T}+\Theta },$$

where $\Theta =T_{w}k_{f}/q_{w}a$ [44]. The global total entropy generation rate can be obtained by integrating Equation (22) over the entire domain Ω. Then an important dimensionless number, Bejan number, representing the relative dominance of entropy generation due to heat transfer and Joule heating [45], is presented as

(24) $$Be=\frac{\int_{\Omega }S_{H}d\Omega }{\int_{\Omega }S_{L}d\Omega }.$$

It is noted that *Be* > 0.5 denotes that heat conduction irreversibility dominates and *Be* < 0.5 denotes the Joule heating irreversibility dominates.

## 3. Numerical Algorithm

Due to the nonlinearity of the governing equations above, the following numerical algorithm is developed to solve the velocity and temperature. Let ${\bar{t}}_{l}=l\Delta \bar{t}$, ${\bar{x}}_{i}=i\Delta \bar{x}$, ${\bar{y}}_{j}=j\Delta \bar{y}$, where $\Delta \bar{t}$ is the temporal step, $\Delta \bar{x}$ and $\Delta \bar{y}$ are the spatial steps with *l* = 1, 2,…, *L*, and *i* = 1, 2, …, *I*, *j* = 1, 2,…, *J*. $f^{l}={\left[f_{i,j}^{l}\right]}_{I\times J}$ represents the matrix obtained by discretizing a variable *f* over the points above. A numerical algorithm is proposed using the time splitting method in terms of time and the compact difference method in space. The complete P-B equation is numerically carried out based on the compact difference schemes, and the corresponding discretized form can be found in [20] and not presented here for conciseness.

To obtain the discretized velocity field ${\bar{w}}^{l}={\left[{\bar{w}}_{i,j}^{l}\right]}_{I\times J}$ with ${\bar{w}}_{i,j}^{l}=\bar{w}({\bar{x}}_{i},{\bar{y}}_{j},{\bar{t}}_{l})$ and the discretized temperature field $T^{l}={\left[{\bar{T}}_{i,j}^{l}\right]}_{I\times J}$ by using the iterative method, one can introduce $\partial /\partial \bar{t}$ on the left-hand side of Equations (11) and (16) and set a nonzero initial value for them,

(25) $$\frac{\partial \bar{w}}{\partial \bar{t}}=\frac{n\eta }{\mu_{0}{\left(1-\phi \right)}^{2.5}}{\left(\frac{W}{a}\right)}^{n-1}\left({\left|\frac{\partial \bar{w}}{\partial \bar{x}}\right|}^{n-1}\frac{\partial^{2}\bar{w}}{\partial {\bar{x}}^{2}}+{\left|\frac{\partial \bar{w}}{\partial \bar{y}}\right|}^{n-1}\frac{\partial^{2}\bar{w}}{\partial {\bar{y}}^{2}}\right)+K^{2}\sinh\bar{\psi },$$

(26) $$\frac{\partial \bar{T}}{\partial \bar{t}}=\frac{\partial^{2}\bar{T}}{\partial {\bar{x}}^{2}}+\frac{\partial^{2}\bar{T}}{\partial {\bar{y}}^{2}}-\frac{k_{f}}{k_{eff}}\left[\left(1+\frac{1}{\alpha }+S\right)\frac{\bar{w}}{{\bar{w}}_{m}}-S\right].$$

More specifically, each time step is split into two steps. In the first step, from ${\bar{t}}_{l}$ to ${\bar{t}}_{*}$, one can focus on the third term on the right-hand side of Equations (19) and (20), and rewrite them as

(27) $$\frac{\partial \bar{w}}{\partial \bar{t}}=K^{2}\sinh\bar{\psi },$$

(28) $$\frac{\partial \bar{T}}{\partial \bar{t}}=-\frac{k_{f}}{k_{eff}}\left[\left(1+\frac{1}{\alpha }+S\right)\frac{\bar{w}}{{\bar{w}}_{m}}-S\right].$$

Accordingly, the velocity field at $\bar{t}={\bar{t}}_{*}$ is given as ${\bar{w}}^{*}={\left[{\bar{w}}_{i,j}^{*}\right]}_{I\times J}={\left[K^{2}\sinh\psi_{i,j}\right]}_{I\times J}$. To obtain temperature field $T^{*}$ at $\bar{t}={\bar{t}}_{*}$, Equation (22) is discretized as

(29) $${\left[\frac{\partial \bar{T}}{\partial \bar{t}}\right]}_{i,j}^{l}=c_{1}c^{l}+c_{2},$$

where $c_{1}=-\frac{k_{f}}{k_{eff}}\left(1+\frac{1}{\alpha }+S\right)$, $c_{2}=\frac{k_{f}}{k_{eff}}S$, and $c^{l}={\left[{\bar{w}}_{i,j}^{l}/{\left(w_{i,j}^{l}\right)}_{m}\right]}_{I\times J}$. The subscript *m* implies the mean value of $w_{i,j}^{l}$. Setting $d^{l}=c_{1}c^{l}+c_{2}$ and the discretized temperature at $\bar{t}={\bar{t}}_{l}$ as $T^{l}$, the Runge–Kutta method is applied to Equation (23):

(30) $$T^{\left(1\right)}=\alpha_{1}T^{l}+\beta_{1}\Delta \bar{t}d^{l},\;T^{\left(2\right)}=\alpha_{2}T^{l}+\beta_{2}[T^{\left(1\right)}+\Delta \bar{t}d^{l}],\;T^{*}=\alpha_{3}T^{l}+\beta_{3}[T^{\left(2\right)}+\Delta \bar{t}d^{l}],$$

where *α*_1_ = *β*_1_ = 1, *α*_2_ = 3/4, *β*_2_ = 1/4, *α*_3_ = 1/3, *β*_2_ = 2/3.

In the second step, from ${\bar{t}}_{*}$ to ${\bar{t}}_{l+1}$, one can focus on the second-order derivatives in Equations (19) and (20), which are generally expressed as

(31) $$\frac{\partial f}{\partial \bar{t}}=C_{1}\frac{\partial^{2}f}{\partial {\bar{x}}^{2}}+C_{2}\frac{\partial^{2}f}{\partial {\bar{y}}^{2}}.$$

To note, as solving ${\bar{w}}^{l}{}_{i,j}$, $C_{1}=n\eta /[\mu_{0}{\left(1-\phi \right)}^{2.5}]{\left(W/a\right)}^{n-1}{\left|\partial \bar{w}/\partial \bar{x}\right|}^{n-1}$ and $C_{2}=n\eta /[\mu_{0}{\left(1-\phi \right)}^{2.5}]{\left(W/a\right)}^{n-1}{\left|\partial \bar{w}/\partial \bar{y}\right|}^{n-1}$, the corresponding discretized forms have been given in Appendix B. When solving ${\bar{T}}^{l}{}_{i,j}$, *C*_1_ = 1 and *C*_2_ = 1. According to the Crank–Nicolson technique, the discretized form of Equation (25) is

(32) $$\begin{array}{l} \frac{f^{l+1}-f^{*}}{\Delta t}-\frac{1}{2}\left\{{\left[C_{1}\frac{\partial^{2}f}{\partial {\bar{x}}^{2}}\right]}^{l+1}+{\left[C_{1}\frac{\partial^{2}f}{\partial {\bar{x}}^{2}}\right]}^{*}\right\}\\ -\frac{1}{2}\left\{{\left[C_{2}\frac{\partial^{2}f}{\partial {\bar{y}}^{2}}\right]}^{l+1}+{\left[C_{2}\frac{\partial^{2}f}{\partial {\bar{y}}^{2}}\right]}^{*}\right\}=O(\Delta t^{2}) \end{array}$$

Using the compact difference schemes and following the procedure presented in Appendix B, *f_i,j_*^l+1^ can be obtained as

(33) $$A_{1}{\vec{P}}_{j-1}^{l+1}+A_{0}{\vec{P}}_{j}^{l+1}+A_{1}{\vec{P}}_{j+1}^{l+1}={Β}_{1}{\vec{P}}_{j-1}^{*}+{Β}_{0}{\vec{P}}_{j}^{*}+{Β}_{1}{\vec{P}}_{j+1}^{*},j=2,3,\cdots ,J-1,$$

where ${\vec{P}}_{j}^{l}={\left(f_{1,j}^{l},f_{2,j}^{l},\ldots ,f_{I,j}^{l}\right)}^{T}$, $A_{0}=tridiag\left(a_{1}-2a_{3},1-2a_{1}-2a_{2}+4a_{3},a_{1}-2a_{3}\right)$, $A_{1}=tridiag\left(a_{3},a_{2}-2a_{3},a_{3}\right)$, ${Β}_{0}=tridiag\left(b_{1}-2b_{3},1-2b_{1}-2b_{2}+4b_{3},b_{1}-2b_{3}\right)$, ${Β}_{1}=tridiag\left(b_{3},b_{2}-2b_{3},b_{3}\right)$, and $h_{\bar{x}}=\frac{\Delta \bar{t}}{\Delta {\bar{x}}^{2}}$, $h_{\bar{y}}=\frac{\Delta \bar{t}}{\Delta {\bar{y}}^{2}}$, $a_{1}=\frac{1}{12}-\frac{C_{1}h_{\bar{x}}}{2}$, $a_{2}=\frac{1}{12}-\frac{C_{2}h_{\bar{y}}}{2}$, $a_{3}=\frac{1}{144}-\frac{C_{1}h_{\bar{x}}}{24}-\frac{C_{2}h_{\bar{y}}}{24}$, $b_{1}=\frac{1}{12}+\frac{C_{1}h_{\bar{x}}}{2}$, $b_{2}=\frac{1}{12}+\frac{C_{2}h_{\bar{y}}}{2}$, $b_{3}=\frac{1}{144}+\frac{C_{1}h_{\bar{x}}}{24}+\frac{C_{2}h_{\bar{y}}}{24}$.

As a result, replacing *f* with $\bar{w}$ and $\bar{T}$, the discretized solutions ${\bar{w}}^{l}{}_{i,j}$ and ${\bar{T}}^{l}{}_{i,j}$ can be computed. Finally, a specified criterion ΔL is given to identify that if the velocity is fully developed, i.e., $\left\|{\bar{w}}_{}^{l}-{\bar{w}}_{}^{l+1}\right\|<\Delta L$. Then, the fully developed velocity ${\bar{w}}^{}{}_{i,j}$ and temperature ${\bar{T}}^{}{}_{i,j}$ are obtained based on the iterative method above.

## 4. Results

A parametric study for velocity, temperature, and Nusselt number is conducted in the case of different flow behavior index *n*, electrokinetic width *K*, volume fraction of nanoparticles *ϕ*, and Joule heating parameter *S*. The nanoparticle is regarded as aluminum oxide [38]. The other physical parameters of the base fluid come from [9] and [20]. The typical values are presented in Table 1 below.

**Figure 2:**
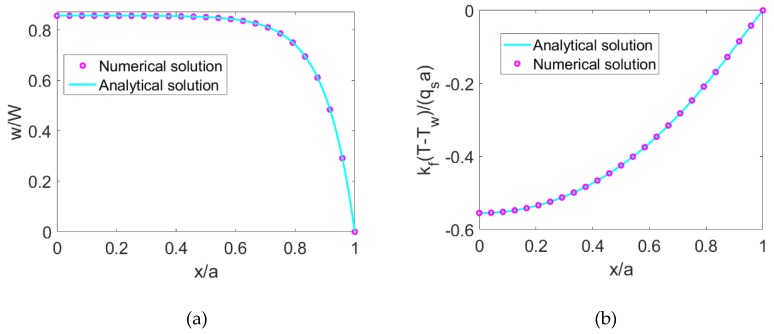
The comparison between the numerical solution and analytical solution from for (**a**) velocity distribution at $\bar{y}=0$ and (**b**) temperature distribution at $\bar{y}=0$.

For validating the numerical algorithm proposed above, a test of grid dependence is conducted. As a result, the volumetric domain *Ω* is discretized to a grid system of 121 × 121. The numeric algorithm shows that the numerical solutions can achieve the accuracy of $O(\Delta {\bar{t}}^{2}+\Delta {\bar{x}}^{4}+\Delta {\bar{y}}^{4})$, which is demonstrated in numerical computation by obtaining the numerical error of the order 10^−8^ between the numerical velocity and analytical velocity of Newtonian nanofluid EOF. Furthermore, the iterative criterion is given as *ΔL* < 10^−10^ to make sure the EOF is fully developed. For the EOF of Newtonian nanofluid, in Figure 2a, the numerical velocity profile at $\bar{y}=0$ is compared with the analytical velocity obtained from Equation (13) and the numerical temperature profile at $\bar{y}=0$ is compared with the analytical temperature obtained from Equation (19) when *K* = 10, *ϕ* = 0.06, and *S* = 3. To clearly show the comparison, 25 grid points of the numerical solution are plotted in Figure 2. It shows that the numerical solutions show good agreement with the analytical solutions and thus, the numerical algorithm is valid, which can be used to numerically solve velocity, temperature, Nusselt number, and Bejan number of power-law nanofluid EOF.

**Figure 3:**
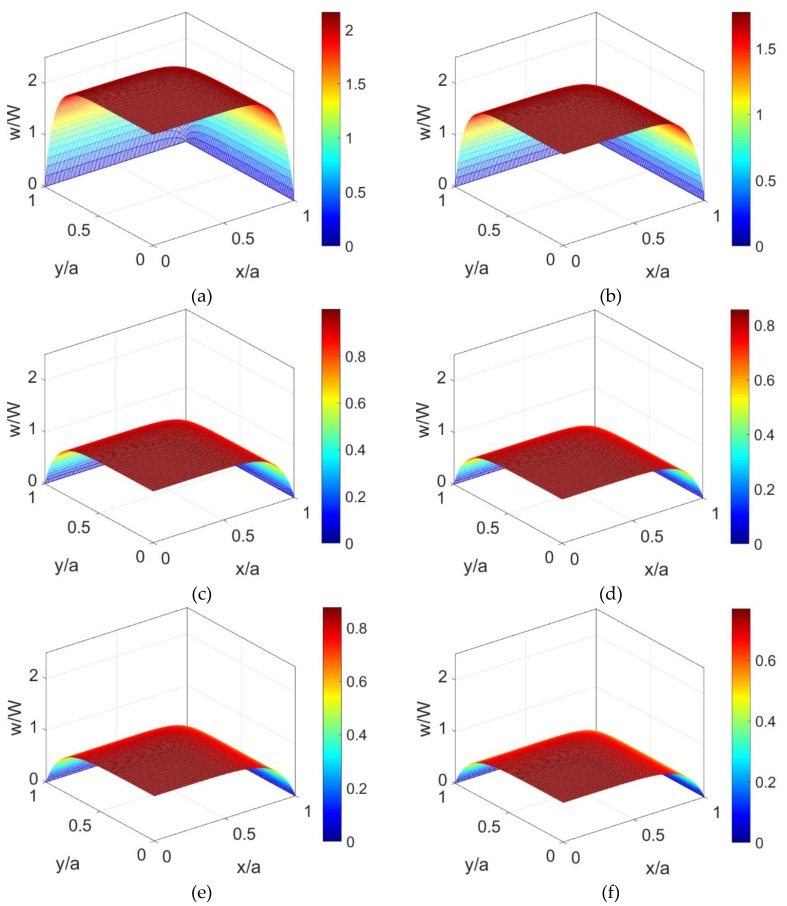
The comparison among the velocity distributions of shear thinning nanofluid, Newtonian nanofluid and shear thickening nanofluid at different nanoparticle volume fraction: (**a**) *ϕ* = 0, *n* = 0.8; (**b**) *ϕ* = 0.06, *n* = 0.8; (**c**) *ϕ* = 0, *n* = 1.0; (**d**) *ϕ* = 0.06, *n* = 1.0; (**e**) *ϕ* = 0, *n* = 1.2; (**f**) *ϕ* = 0.06, *n* = 1.2.

Figure 3 illustrates velocity distributions of shear thickening nanofluid, Newtonian nanofluid, and shear thinning nanofluid at different volume fractions when *K* = 10. Figure 3a,c,e show the velocity distributions of shear thinning fluid, Newtonian fluid, and shear thickening fluid without consideration of nanoparticles in fluid, namely at *ϕ* = 0. Figure 3b,d,f show the velocity distributions of shear thinning nanofluid, Newtonian nanofluid, and shear thickening nanofluid at *ϕ* = 0.06. It is noted from these two groups of figures that the velocity shows homogeneous abatement across the channel as the volume fraction of nanoparticles increases no matter what fluid rheology is considered. This is because the nanoparticles in fluid enhance the effective viscosity of power-law nanofluid in response to the sear rate and cause greater dispersion of the velocity distribution. As observed in [7], although considering the EOF of power-law nanofluid, the shear thinning feature of base fluid leads to greater velocity compared to the shear thickening feature of the base fluid.

**Figure 4:**
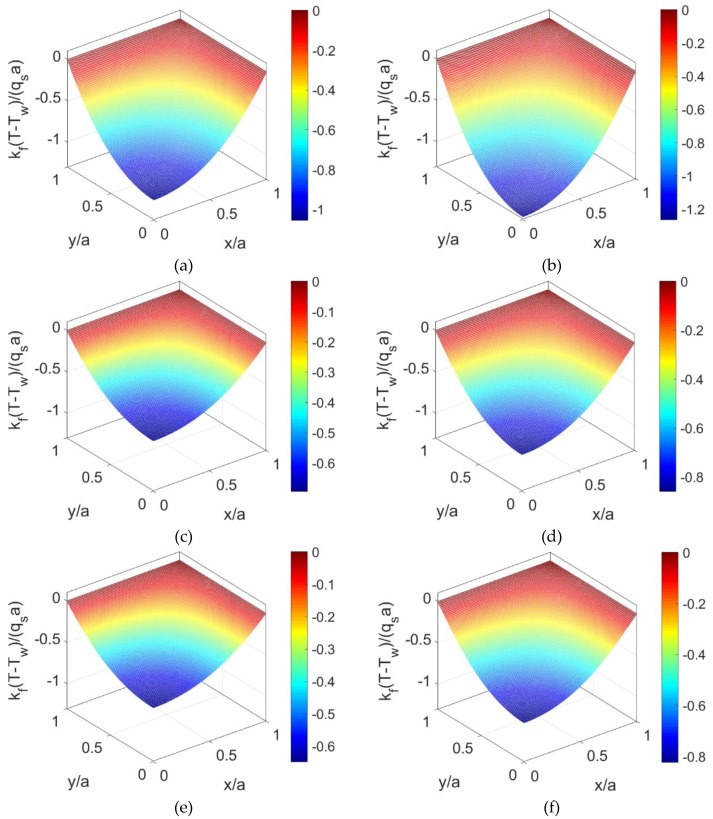
The dimensionless temperature distributions of shear thinning fluid, Newtonian fluid, and shear thickening fluid for different Joule heating parameter when nanoparticle volume fraction is *ϕ* = 0: (**a**) *n* = 0.8, *S* = 0; (**b**) *n* = 0.8, *S* = 3; (**c**) *n* = 1.0, *S* = 0; (**d**) *n* = 1.0, *S* = 3; (**e**) *n* = 1.2, *S* = 0; (**f**) *n* = 1.2, *S* = 3.

Without consideration of nanoparticles in fluid (*ϕ* = 0) and when *K* = 10, the temperature distributions of shear thinning fluid, Newtonian fluid, and shear thickening fluid at *S* = 0 are displayed in Figure 4a,c,e. For the same prescribed conditions, the temperature distributions at *S* = 3 are displayed in Figure 4b,d,f. The comparison between these two groups of figures shows that the variation of temperature along the channel becomes greater with the increase of the Joule heating parameter *S* irrespective of the fluid type. Furthermore, the dimensionless temperature increases with the flow behavior index *n*, indicating that the difference of heat transfer characters caused by different shear rates of fluid type is remarkable, especially for shear thinning base fluid.

**Figure 5:**
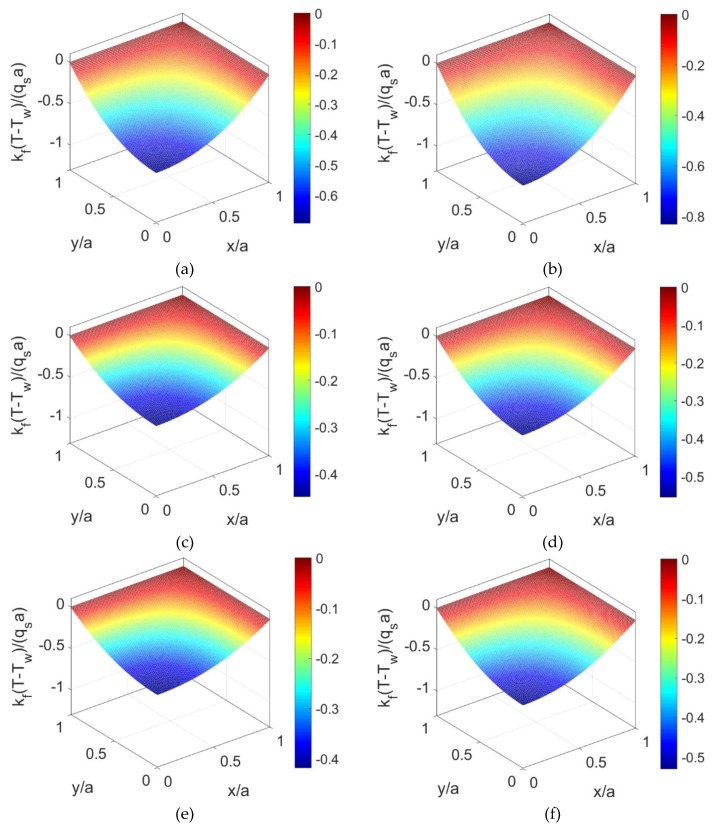
The dimensionless temperature distributions of shear thinning nanofluid, Newtonian nanofluid, and shear thickening nanofluid for different Joule heating parameters *S* when nanoparticle volume fraction is *ϕ* = 0.06: (**a**) *n* = 0.8, *S* = 0; (**b**) *n* = 0.8, *S* = 3; (**c**) *n* = 1.0, *S* = 0; (**d**) *n* = 1.0, *S* = 3; (**e**) *n* = 1.2, *S* = 0; (**f**) *n* = 1.2, *S* = 3.

**Figure 6:**
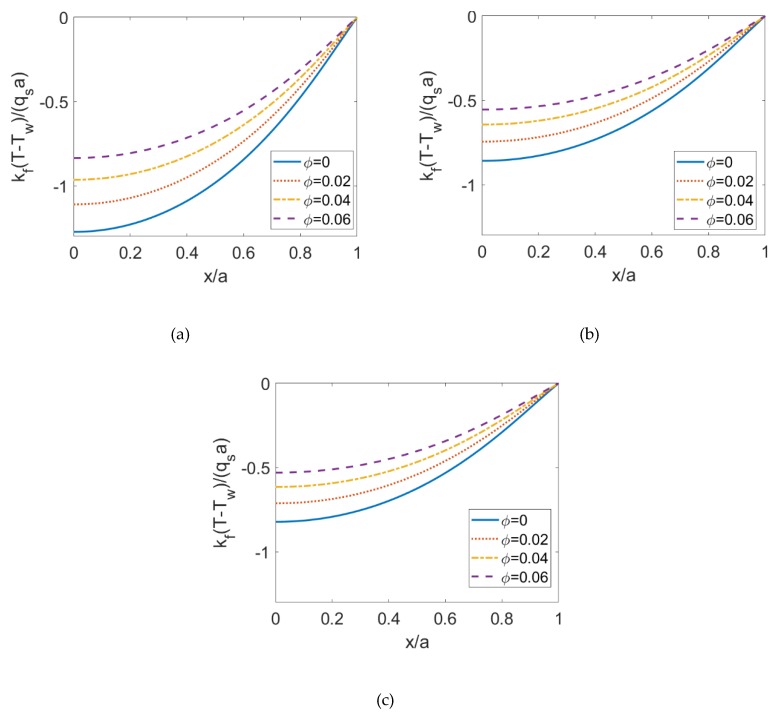
The comparison among temperature profiles at $\bar{y}=0$ for different nanoparticle volume fractions *ϕ* in the case of (**a**) *n* = 0.8, (**b**) *n* = 1.0 and (**c**) *n* = 1.2.

To observe the influence of volume fraction of nanoparticles on the heat transfer characteristics, the temperature distributions are evaluated in Figure 5 when the nanoparticle volume fraction *ϕ* is increased to 0.06, and other parameters keep unchanged. The corresponding temperature profiles at $\bar{y}=0$ is illustrated in Figure 6 for different volume fractions of the nanoparticles. From Figure 6 and the comparison between Figure 4 and Figure 5, the dimensionless temperature distribution shows an increment when the volume fraction of nanoparticles *ϕ* increases, or the flow behavior index *n* increases. It means that the combined effects of larger thermal conductivity of nanoparticles and the change in shear stress leads to the increase in thermal diffusion effect and in further the temperature.

**Figure 7:**
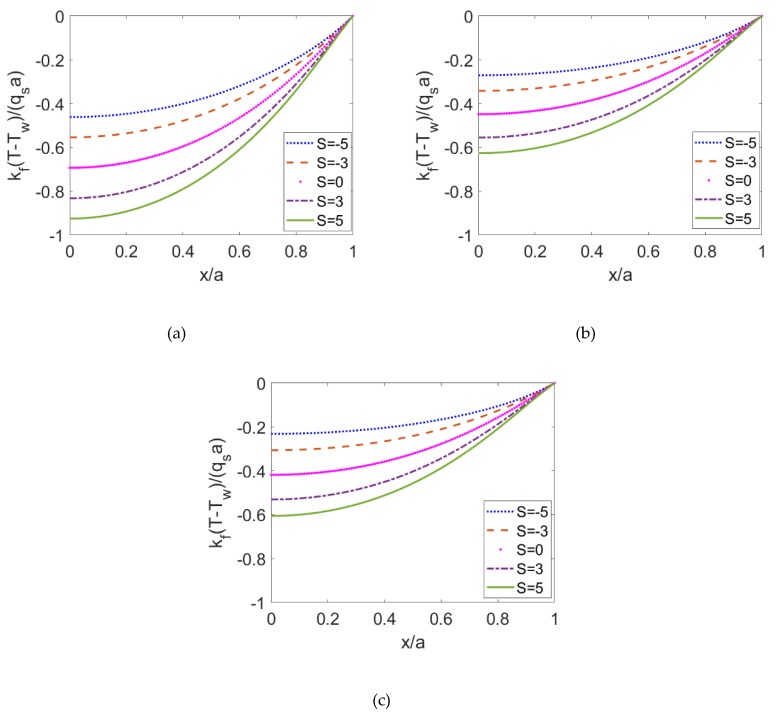
The comparison among temperature profiles at $\bar{y}=0$ for different Joule heating parameters in the case of (**a**) *n* = 0.8, (**b**) *n* = 1.0 and (**c**) *n* = 1.2.

To observe the influence of the Joule heating parameter on the temperature, in Figure 7, the temperature profiles at $\bar{y}=0$ for different Joule heating parameters *S* are illustrated in the case of shear thinning nanofluid, Newtonian nanofluid, and shear thickening nanofluid when *ϕ* = 0.06 and *K* = 10. As seen in Figure 5, no matter what nanofluid is considered, as the Joule heating parameter increases the variation of temperature increases, namely, the temperature difference between the centerline and the channel wall enlarges. It implies that although the energy from Joule heating effect is uniformly enhanced, the heat transferred through convection near the channel wall is much less than near the centerline. And this effect is more notable when considering shear thinning base fluid.

**Figure 8:**
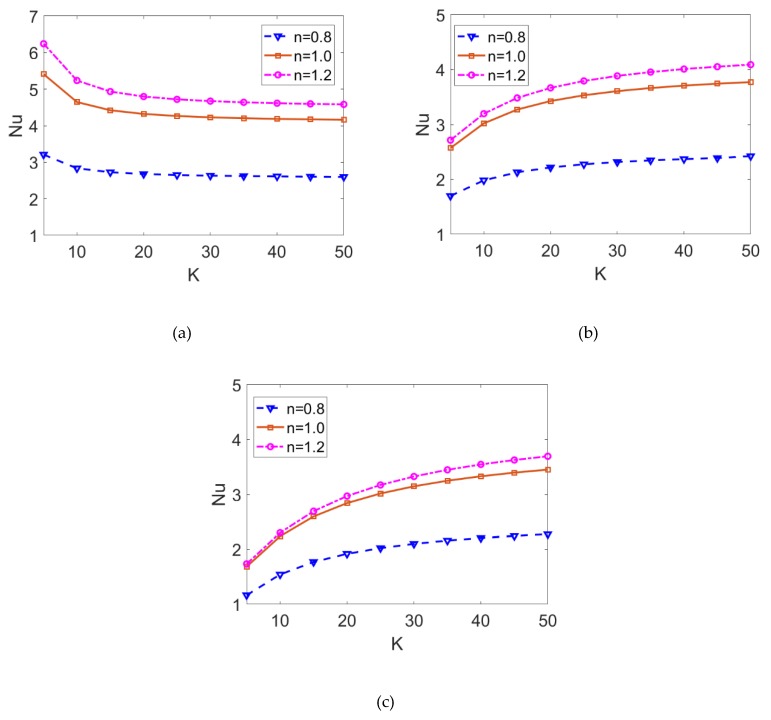
The variation of Nusselt number with the electrokinetic width *K* for shear thinning nanofluid, Newtonian nanofluid, shear thickening nanofluid when the Joule heating parameters are given as (**a**) *S* = −5; (**b**) *S* = 0; (**c**) *S* = 5.

The variations of the Nusselt number with the electrokinetic width are shown in Figure 8 for different fluid types and Joule heating parameters when *ϕ* = 0.06. The cases *S* < 0 and *S* > 0 correspond to the surface cooling effect and surface heating effect, respectively. Importantly, in the case of *S* < 0, the Nusselt number decreases as electrokinetic width *K* increases, which increases when S ≥ 0 and other parameters remain unchanged. Surface heating (cooling) has a uniform influence on the temperature distribution, which leads to the increasing (decreasing) tendency of Nusselt number with electrokinetic width. Furthermore, the decrease in flow behavior index results in the pronounced abatement of Nusselt number, meaning that the heat transfer rate of shear thinning nanofluid is much smaller than the heat transfer rate of Newtonian fluid and shear thickening fluid. This is consistent with the prediction in [22].

**Figure 9:**
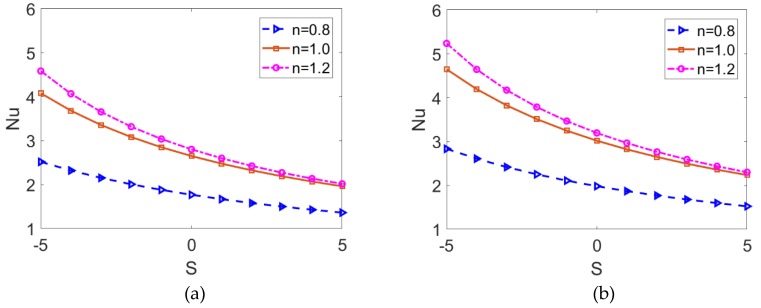
The variation of Nusselt number with Joule heating parameter for shear thinning nanofluid, Newtonian nanofluid, and shear thickening nanofluid in the case of (**a**) *ϕ* = 0 and (**b**) *ϕ* = 0.06.

Figure 9 depicts the variations of the Nusselt number with the Joule heating parameter for different fluid types and different nanoparticle volume fraction when *K* = 10. It shows that the Nusselt number is inversely proportional to the Joule heating parameter, and the decreasing rate becomes smaller as nanofluid changes from shear thickening to shear thinning. Since it is considered that Joule heating has a spatially uniform effect, the greater Joule heating parameter corresponds to relatively uniform heat distribution. Consequently, the convective heat transfer rate is reduced as presented in Figure 7, and one can observe the decreasing trend of the Nusselt number, namely, heat transfer rate. Being consistent with the prediction in Figure 7, the smaller convective heat transfer rate of shear thinning nanofluid leads to a smaller Nusselt number. Furthermore, the comparison between Figure 9a,b show that no matter what base fluid and what Joule heating parameter is taken, the increase in the volume fraction of nanoparticles *ϕ* significantly improves the heat transfer rate.

**Figure 10:**
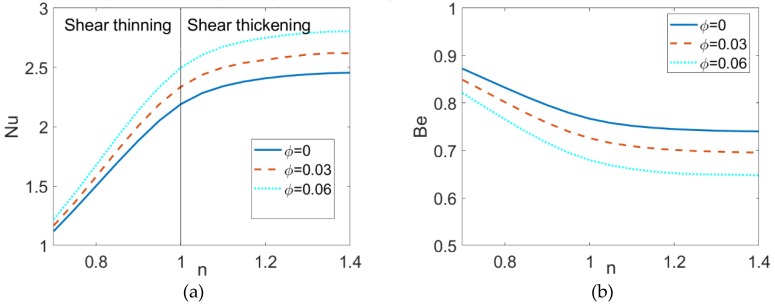
The variation of (**a**) Nusselt number and (**b**) Bejan number with flow behavior index for different nanoparticle volume fraction.

The variation of the Nusselt number *Nu* and the variation of Bejan number *Be* with flow behavior index *n* for different volume fractions of nanoparticles are depicted in Figure 10 when *K* = 20 and *S* = 3. The combined effects of nonlinear rheological behavior and nanoparticle volume fraction on Nusselt number are investigated. To note, the dependence of Nusselt number on the flow behavior index shows nonlinear. For shear thinning base fluid, the Nusselt number is more amenable to the change in flow behavior index *n*. The increase in viscosity for shear thinning fluid resulting from the decrease in flow behavior index reduces the Nusselt number, and thus, the heat transfer rate of shear thinning nanofluid is obviously less than that of shear thickening nanofluid. Furthermore, the variation of the Nusselt number with the volume fraction of nanoparticles is almost linear. It shows that the heat transfer performance of nanofluid is clearly enhanced compared to the conventional fluid regardless of the base fluid rheology. These results are also witnessed in Figure 10b. The increase in nanoparticle volume fraction and flow behavior index leads to the enhancement of heat transfer, which further reduces the “loss” of useful energy, i.e., the total irreversibility of the system. Moreover, the *Be* > 0.5 implies that the primary irreversibility is contributed to the heat conduction when *S* = 3.

## 5. Conclusions

In the present paper, a numerical investigation for hydrodynamically and thermally fully developed EOF of power-law nanofluid in a rectangular microchannel has been conducted. The interactive influences of electrokinetic width *K*, flow behavior index *n*, nanoparticle volume fraction *ϕ,* and Joule heating parameter *S* on the velocity field, temperature field, Nusselt number, and Bejan number have been studied. The following conclusions can be drawn:

- The velocity is reduced due to the increase in viscosity of nanofluid resulting from the addition of nanoparticles.
- The volume fraction of nanoparticles *ϕ* truly enhances the dimensionless temperature and Nusselt number by changing the thermal conductivity and shear stress of velocity, no matter what type of base fluid is considered. Accordingly, the Bejan number decreases with increasing nanoparticle volume fraction and flow behavior index.
- Nusselt number is an increasing function of the electrokinetic width *K* for *S* ≥ 0 and a decreasing function for *S* < 0.
- The Nusselt number shows abatement, and the temperature exhibits greater variation along the channel as Joule heating parameter *S* increases. The heat transfer rate of shear thickening nanofluid is much larger than that of shear thinning fluid.

To sum up, nanoparticle volume fraction and Joule heating effect show significant influence on the thermal behavior of EOF of power-law nanofluid. It should be noted that the dependence of the Nusselt number on the flow behavior index is nonlinear. The Nusselt number becomes a weak function of electrokinetic width as the electrokinetic width gets larger. The heat transfer rate of shear thinning nanofluid is more amenable to the change in nanofluid rheology. Therefore, the heat transfer of power-law nanofluid has to be carefully treated, and one should make a distinction when manipulating different types of nanofluid in practical problems. This understanding of the mechanism of thermally fully developed EOF can be referred to in more efficient control of fluids and optimization of microfluidic devices.

## Figures and Tables

**Table 1:** Typical values of the parameters.

Parameters (Notation)	Value (unit)
The fluid permittivity *ε*	7.08 × 10^−10^ C·V^−1^·m^−1^
Boltzmann constant *k_b_*	1.38 × 10^−23^ J·K^−1^
Absolute temperature *T*_0_	293 K
Elementary charge *e*	1.6 × 10^−19^ C
Half channel height and width, *a* and *b*	10 × 10^−6^ m
Fluid electrical conductivity *σ*	1.2639 × 10^−7^ S·m^−1^
Flow consistency index of power-law fluid *η*	9 × 10^−4^ N·m*^−^*^2^·s*^n^*
Viscosity of Newtonian fluid *μ*_0_	9 × 10^−4^ N·m^−2^·s
Zeta potential *ξ*	0.025 V
Thermal conductivity of the solid nanoparticle *k_s_*	40 W·m^−1^·K^−1^
Thermal conductivity of the base fluid *k_f_*	0.618 W·m^−1^·K^−1^
Valence of ions *χ*	1
Electrokinetic width *K*	5–50
Flow behavior index *n*	0.7–1.4
Nanoparticle volume fraction *ϕ*	0.0–0.06
Joule heating Parameter *S*	−5–5
Ratio of nanolayer thickness to original particle radius *ω*	1.1

## Appendix A

The velocity distribution of the Newtonian nanofluid is solved following the procedure in [40] and is presented as Equation (13). As the nanoparticle volume fraction vanishes, the velocity of Equation (13) becomes Equation (14) in [21].

The temperature distribution of Newtonian nanofluid EOF is solved as follows: in the first step, based on the method of variable separation [40], the temperature can be expressed as

(A1) $$\bar{T}(\bar{x},\bar{y})=\sum_{I=1}^{\infty }\cos(\sqrt{\beta_{I}}\bar{x})Y_{I}(\bar{y})$$

with $\beta_{I}=(2I-1)\pi /(2\alpha )$, *I* = 1,2,3,….

In the second step, $Y_{I}(\bar{y})$ needs to be determined. Substituting Equation (A1) into Equation (17) yields

(A1a) $$\sum_{I=1}^{\infty }\cos(\sqrt{\beta_{I}}\bar{x})\chi_{I}(\bar{y})=k_{1}\bar{w}-k_{2}S,$$

(A1b) $$\left[\frac{d^{2}Y_{I}(\bar{y})}{d{\bar{y}}^{2}}-\beta_{I}Y_{I}(\bar{y})\right]=\chi_{I}(\bar{y}).$$

$\chi_{I}(\bar{y})$ can be expressed from Equation (A1a) using the orthogonality of Fourier series, namely,

(A2) $$\chi_{I}(\bar{y})=\frac{2}{\alpha }\int_{0}^{\alpha }\left[k_{1}\bar{w}-k_{2}S\right]\cos(\sqrt{\beta_{I}}\bar{x})d\bar{x},$$

where $k_{1}=\frac{k_{f}}{k_{eff}}\left(1+\frac{1}{\alpha }+S\right)$ and $k_{2}=\frac{k_{f}}{k_{eff}}$. Replacing $\bar{w}$ with Equation (13), further rearrangements produce $\chi_{I}(\bar{y})=k_{1}\left[U_{I}(\bar{y})\right]-B_{I}$, where $B_{I}=2k_{2}S{\left(-1\right)}^{I+1}/(\alpha \sqrt{\beta_{I}})$.

In the third step, with the expression of $\chi_{I}(\bar{y})$, the ODE of Equation (A1b) is solved adopting the method of constant variation, the solution form is given as

(A3) $$Y_{I}(\bar{y})=Y_{IG}(\bar{y})+Y_{IP}(\bar{y}),$$

(A3a) $$Y_{IG}(\bar{y})=H_{1}e^{C_{I}\bar{y}}+H_{2}e^{-C_{I}\bar{y}},$$

(A3b) $$Y_{IP}(\bar{y})=\frac{1}{C_{I}}\int_{0}^{\bar{y}}\chi_{I}(s)\sinh[C_{I}(\bar{y}-s)]ds.$$

Here Equation (A3a) indicates the general solution of the homogeneous version of Equation (A1b). Equation (A3b) indicates the particular solution of Equation (A1b), which is determined by the method of Laplace transformation. Combining boundary condition, the coefficients are deduced as

(A4) $$H_{1}=H_{2}=-\frac{1}{2\cosh(C_{I})}\frac{1}{C_{I}}\int_{0}^{1}\chi_{I}(s)\sinh[C_{I}(1-s)]ds.$$

As a result, Equation (A3) becomes

(A5) $$\begin{array}{cc} Y_{I}(\bar{y})=- & \frac{\cosh(C_{I}\bar{y})}{\cosh(C_{I})}\frac{1}{C_{I}}\left[\int_{0}^{1}k_{1}\left[U_{I}(s)\right]\sinh[C_{I}(1-s)]ds+\frac{B_{I}}{C_{I}}\left(1-\cosh C_{I}\right)\right],\\ & +\frac{B_{I}}{C_{I}{}^{2}}[1-\cosh(C_{I}\bar{y})]+\frac{k_{1}}{C_{I}}\int_{0}^{\bar{y}}\left(U_{I}(s)\right)\sinh[C_{I}(\bar{y}-s)]ds. \end{array}$$

Substituting the expression of $U_{I}(y)$ into Equation (A5) yields

(A6) $$Y_{I}(\bar{y})=-L_{I}^{\left(0\right)}\{\frac{-k_{1}AL_{I}{}^{\left(1\right)}}{L_{I}^{\left(0\right)}}\left(\sum_{M=1}^{\infty }\frac{A_{MI}G_{MI}}{D_{M}}+\sum_{N=1}^{\infty }\frac{B_{NI}Q_{NI}}{E_{N}}\right)\;+k_{1}A\sum_{M=1}^{\infty }\frac{A_{MI}(L_{I}{}^{\left(1\right)}-L_{MI}{}^{\left(2\right)})}{D_{M}(\mu_{M}{}^{2}+C_{I}{}^{2})}+k_{1}A\sum_{N=1}^{\infty }\frac{B_{NI}(L_{NI}{}^{\left(3\right)}-L_{I}{}^{\left(1\right)})}{E_{N}(\sigma_{N}{}^{2}+K^{2}-C_{I}{}^{2})}+\frac{B_{I}}{C_{I}}\left(1-\cosh C_{I}\right)\}\cosh(C_{I}\bar{y})\;+\frac{B_{I}}{C_{I}{}^{2}}[1-\cosh(C_{I}\bar{y})]-\frac{k_{1}A}{C_{I}L_{I}^{\left(0\right)}}\left(\sum_{M=1}^{\infty }\frac{A_{MI}G_{MI}}{D_{M}}+\sum_{N=1}^{\infty }\frac{B_{NI}Q_{NI}}{E_{N}}\right)\frac{\bar{y}\sinh(C_{I}\bar{y})}{2}\;+\frac{k_{1}A}{C_{I}}\sum_{M=1}^{\infty }\frac{A_{MI}}{D_{M}(\mu_{M}{}^{2}+C_{I}{}^{2})}\left\{\frac{\bar{y}\sinh(C_{I}\bar{y})}{2}+\frac{C_{I}[\cos(\mu_{M}\bar{y})-\cosh(C_{I}\bar{y})]}{C_{I}{}^{2}+\mu_{M}{}^{2}}\right\}\;+\frac{k_{1}A}{C_{I}}\sum_{N=1}^{\infty }\frac{B_{NI}}{E_{N}(\sigma_{N}{}^{2}+K^{2}-C_{I}{}^{2})}\left\{\frac{C_{I}[\cosh(\sqrt{\sigma_{N}{}^{2}+K^{2}}\bar{y})-\cosh(C_{I}\bar{y})]}{\sigma_{N}{}^{2}+K^{2}-C_{I}{}^{2}}-\frac{\bar{y}\sinh(C_{I}\bar{y})}{2}\right\}$$

where $L_{I}{}^{\left(0\right)}=\frac{1}{C_{I}\cosh(C_{I})}$, $L_{I}{}^{\left(1\right)}=\int_{0}^{1}\cosh(C_{I}s)\sinh[C_{I}(1-s)]ds=\frac{1}{2}\sinh(C_{I})$,

$L_{MI}{}^{\left(2\right)}=\int_{0}^{1}\cos(\mu_{M}y)\sinh[C_{I}(1-s)]ds=-\frac{C_{I}[\cos(\mu_{M})-\cosh(C_{I})]}{C_{I}{}^{2}+\mu_{M}{}^{2}}$,

$L_{NI}{}^{\left(3\right)}=\int_{0}^{1}\cosh(\sqrt{\sigma_{N}{}^{2}+K^{2}}s)\sinh[C_{I}(1-s)]ds=\frac{C_{I}[\cosh(\sqrt{\sigma_{N}{}^{2}+K^{2}})-\cosh(C_{I})]}{\sigma_{N}{}^{2}+K^{2}-C_{I}{}^{2}}.$

Consequently, the analytical solution of temperature for Newtonian nanofluid flow is carried out.

## Appendix B

In the case of $f=\bar{w}$, the compact difference schemes for computing *C*_1_ and *C*_2_ are given as

(B1) $$\frac{1}{6}F_{\bar{x},i+1,j}\left(\bar{w}\right)+\frac{2}{3}F_{\bar{x},i,j}\left(\bar{w}\right)+\frac{1}{6}F_{\bar{x},i-1,j}\left(\bar{w}\right)=({\bar{w}}_{i+1,j}-{\bar{w}}_{i-1,j})/2\Delta \bar{x},$$

(B2) $$\frac{1}{6}F_{\bar{y},i,j+1}\left(\bar{w}\right)+\frac{2}{3}F_{\bar{y},i,j}\left(\bar{w}\right)+\frac{1}{6}F_{\bar{y},i,j-1}\left(\bar{w}\right)=({\bar{w}}_{i,j+1}-{\bar{w}}_{i,j-1})/2\Delta \bar{y},$$

in which $F_{\bar{x},i,j}\left(\bar{w}\right)$ and $F_{\bar{y},i,j}\left(\bar{w}\right)$ are the difference approximations of $\partial \bar{w}/\partial \bar{x}$ and $\partial \bar{w}/\partial \bar{y}$, respectively.

To derive the further discretized form of Equation (32), setting $\delta_{\bar{x}}^{2}f_{i,j}=\left(f_{i+1,j}-2f_{i,j}+f_{i-1,j}\right)/\Delta {\bar{x}}^{2}$, $\delta_{\bar{y}}^{2}f_{i,j}=\left(f_{i,j+1}-2f_{i,j}+f_{i,j-1}\right)/\Delta {\bar{y}}^{2}$, the following finite difference operators are introduced:

(B3) $${\bar{\delta }}_{\bar{x}}^{2}f_{i,j}=\left(1+\frac{\Delta {\bar{x}}^{2}}{12}\delta_{\bar{x}}^{2}\right)f_{i,j},\;{\bar{\delta }}_{\bar{y}}^{2}f_{i,j}=\left(1+\frac{\Delta {\bar{y}}^{2}}{12}\delta_{\bar{y}}^{2}\right)f_{i,j}.$$

Note that,

(B4) $$\delta_{\bar{x}}^{2}f_{i,j}={\bar{\delta }}_{\bar{x}}^{2}{\left[\frac{\partial^{2}f}{\partial {\bar{x}}^{2}}\right]}_{i,j}+O(\Delta {\bar{x}}^{4}),\delta_{\bar{y}}^{2}f_{i,j}={\bar{\delta }}_{\bar{y}}^{2}{\left[\frac{\partial^{2}f}{\partial {\bar{y}}^{2}}\right]}_{i,j}+O(\Delta {\bar{y}}^{4})$$

or symbolically,

(B5) $${\bar{\delta }}_{\bar{x}}^{-2}\delta_{\bar{x}}^{2}f_{i,j}={\left[\frac{\partial^{2}f}{\partial {\bar{x}}^{2}}\right]}_{i,j}+O(\Delta {\bar{x}}^{4}),\;{\bar{\delta }}_{\bar{y}}^{-2}\delta_{\bar{y}}^{2}f_{i,j}={\left[\frac{\partial^{2}f}{\partial {\bar{x}}^{2}}\right]}_{i,j}+O(\Delta {\bar{y}}^{4}),$$

where ${\bar{\delta }}_{\bar{x}}^{-2}={\left({\bar{\delta }}_{\bar{x}}^{2}\right)}^{-1}$ and ${\bar{\delta }}_{\bar{y}}^{-2}={\left({\bar{\delta }}_{\bar{y}}^{2}\right)}^{-1}$ are the inverse of ${\bar{\delta }}_{\bar{x}}^{2}$ and ${\bar{\delta }}_{\bar{y}}^{2}$, respectively.

The fourth-order compact approximations (B5) is applied to the derivatives in Equation (32), and it yields:

(B6) $$\frac{f^{l+1}-f^{*}}{\Delta \bar{t}}-\frac{C_{1}}{2}\left({\bar{\delta }}_{\bar{x}}^{-2}{\bar{\delta }}_{\bar{x}}^{2}f_{i,j}^{l+1}+{\bar{\delta }}_{\bar{x}}^{-2}{\bar{\delta }}_{\bar{x}}^{2}f_{i,j}^{*}\right)-\frac{C_{2}}{2}\left({\bar{\delta }}_{\bar{y}}^{-2}{\bar{\delta }}_{\bar{y}}^{2}f_{i,j}^{l+1}+{\bar{\delta }}_{\bar{y}}^{-2}{\bar{\delta }}_{\bar{y}}^{2}f_{i,j}^{*}\right)=O(\Delta {\bar{t}}^{2}+\Delta {\bar{x}}^{4}+\Delta {\bar{y}}^{4}).$$

Multiplying the above equation by the finite difference operator ${\bar{\delta }}_{\bar{x}}^{2}{\bar{\delta }}_{\bar{y}}^{2}$, one has

(B7) $$\left({\bar{\delta }}_{\bar{x}}^{2}{\bar{\delta }}_{\bar{y}}^{2}-\frac{\Delta \bar{t}C_{1}}{2}{\bar{\delta }}_{\bar{y}}^{2}\delta_{\bar{x}}^{2}-\frac{\Delta \bar{t}C_{1}}{2}{\bar{\delta }}_{\bar{x}}^{2}\delta_{\bar{y}}^{2}\right)f_{i,j}^{l+1}\;=\left({\bar{\delta }}_{\bar{x}}^{2}{\bar{\delta }}_{\bar{y}}^{2}+\frac{\Delta \bar{t}C_{1}}{2}{\bar{\delta }}_{\bar{y}}^{2}\delta_{\bar{x}}^{2}+\frac{\Delta \bar{t}C_{1}}{2}{\bar{\delta }}_{\bar{x}}^{2}\delta_{\bar{y}}^{2}\right)f_{i,j}^{*}+O\left(\Delta {\bar{t}}^{3}+\Delta \bar{t}\Delta {\bar{x}}^{4}+\Delta \bar{t}\Delta {\bar{y}}^{4}\right).$$

Rearranging Equation (B7) and dropping the term $O\left(\Delta {\bar{t}}^{3}+\Delta \bar{t}\Delta {\bar{x}}^{4}+\Delta \bar{t}\Delta {\bar{y}}^{4}\right)$, the left-hand side and the right-hand side can be approximated, respectively, as

(B8) $$\begin{array}{c} \left({\bar{\delta }}_{\bar{x}}^{2}{\bar{\delta }}_{\bar{y}}^{2}-\frac{\Delta \bar{t}C_{1}}{2}{\bar{\delta }}_{\bar{y}}^{2}\delta_{\bar{x}}^{2}-\frac{\Delta \bar{t}C_{1}}{2}{\bar{\delta }}_{\bar{x}}^{2}\delta_{\bar{y}}^{2}\right)f_{i,j}^{l+1}=\left(1-2a_{1}-2a_{2}+4a_{3}\right)f_{i,j}^{l+1}\\ +\left(a_{1}-2a_{3}\right)\left(f_{i+1,j}^{l+1}+f_{i-1,j}^{l+1}\right)+\left(a_{2}-2a_{3}\right)\left(f_{i,j+1}^{l+1}+f_{i,j-1}^{l+1}\right)\\ +a_{3}\left(f_{i+1,j+1}^{l+1}+f_{i+1,j-1}^{l+1}+f_{i-1,j+1}^{l+1}+f_{i-1,j-1}^{l+1}\right) \end{array}$$

(B9) $$\begin{array}{c} \left({\bar{\delta }}_{\bar{x}}^{2}{\bar{\delta }}_{\bar{y}}^{2}+\frac{\Delta \bar{t}C_{1}}{2}{\bar{\delta }}_{\bar{y}}^{2}\delta_{\bar{x}}^{2}+\frac{\Delta \bar{t}C_{1}}{2}{\bar{\delta }}_{\bar{x}}^{2}\delta_{\bar{y}}^{2}\right)f_{i,j}^{*}=\left(1-2b_{1}-2b_{2}+4b_{3}\right)f_{i,j}^{*}\\ +\left(b_{1}-2b_{3}\right)\left(f_{i+1,j}^{*}+f_{i-1,j}^{*}\right)+\left(b_{2}-2b_{3}\right)\left(f_{i,j+1}^{*}+f_{i,j-1}^{*}\right)\\ +b_{3}\left(f_{i+1,j+1}^{*}+f_{i+1,j-1}^{*}+f_{i-1,j+1}^{*}+f_{i-1,j-1}^{*}\right) \end{array}$$

with $h_{\bar{x}}=\Delta \bar{t}/\Delta {\bar{x}}^{2}$, $h_{\bar{y}}=\Delta \bar{t}/\Delta {\bar{y}}^{2}$, $a_{1}=1/12-C_{1}h_{\bar{x}}/2$, $a_{2}=1/12-C_{2}h_{\bar{y}}/2$, $b_{1}=1/12+C_{1}h_{\bar{x}}/2$, $b_{2}=1/12+C_{2}h_{\bar{y}}/2$, $a_{3}=1/144-C_{1}h_{\bar{x}}/24-C_{2}h_{\bar{y}}/24$, $b_{3}=1/144+C_{1}h_{\bar{x}}/24+C_{2}h_{\bar{y}}/24$.

The following second-order one-side difference approximations are applied to the boundary conditions expressed as Equations (12) and (18):

(B10) $${\left[\frac{\partial^{2}f}{\partial {\bar{x}}^{2}}\right]}_{1,j}=\left(2f_{1,j}-5f_{2,j}+4f_{3,j}-f_{4,j}\right)/\Delta {\bar{x}}^{2},\;{\left[\frac{\partial^{2}f}{\partial {\bar{x}}^{2}}\right]}_{I,j}=\left(2f_{I,j}-5f_{I-1,j}+4f_{I-2,j}-f_{I-3,j}\right)/\Delta {\bar{x}}^{2},\;{\left[\frac{\partial^{2}f}{\partial {\bar{y}}^{2}}\right]}_{i,1}=\left(2f_{i,1}-5f_{i,2}+4f_{i,3}-f_{i,4}\right)/\Delta {\bar{y}}^{2},\;{\left[\frac{\partial^{2}f}{\partial {\bar{y}}^{2}}\right]}_{i,J}=\left(2f_{i,J}-5f_{i,J-1}+4f_{i,J-2}-f_{i,J-3}\right)/\Delta {\bar{y}}^{2}.$$

Consequently, the discretized form of *f* can be expressed as a column vector ${\vec{P}}_{j}^{l}={\left(f_{1,j}^{l},f_{2,j}^{l},\ldots ,f_{I,j}^{l}\right)}^{T}$, and the corresponding coefficients can be presented as the symmetric tridiagonal matrices given in Equation (33).
